# Central spindle proteins and mitotic kinesins are direct transcriptional targets of MuvB, B-MYB and FOXM1 in breast cancer cell lines and are potential targets for therapy

**DOI:** 10.18632/oncotarget.14466

**Published:** 2017-01-03

**Authors:** Patrick Wolter, Steffen Hanselmann, Grit Pattschull, Eva Schruf, Stefan Gaubatz

**Affiliations:** ^1^ Theodor Boveri Institute, Biocenter, University of Wuerzburg and Comprehensive Cancer Center Mainfranken, University of Wuerzburg, University of Wuerzburg, Wuerzburg, Germany

**Keywords:** B-MYB, FOXM1, MuvB, DREAM, kinesin

## Abstract

The MuvB multiprotein complex, together with B-MYB and FOXM1 (MMB-FOXM1), plays an essential role in cell cycle progression by regulating the transcription of genes required for mitosis and cytokinesis. In many tumors, B-MYB and FOXM1 are overexpressed as part of the proliferation signature. However, the transcriptional targets that are important for oncogenesis have not been identified. Given that mitotic kinesins are highly expressed in cancer cells and that selected kinesins have been reported as target genes of MMB-FOXM1, we sought to determine which mitotic kinesins are directly regulated by MMB-FOXM1. We demonstrate that six mitotic kinesins and two microtubule-associated non-motor proteins (MAPs) CEP55 and PRC1 are direct transcriptional targets of MuvB, B-MYB and FOXM1 in breast cancer cells. Suppression of KIF23 and PRC1 strongly suppressed proliferation of MDA-MB-231 cells. The set of MMB-FOXM1 regulated kinesins genes and 4 additional kinesins which we referred to as the mitotic kinesin signature (MKS) is linked to poor outcome in breast cancer patients. Thus, mitotic kinesins could be used as prognostic biomarker and could be potential therapeutic targets for the treatment of breast cancer.

## INTRODUCTION

Cell cycle regulation is essential for normal development and, when disturbed, can lead to a number of diseases such as cancer. MuvB is an evolutionary conserved multisubunit complex that regulates gene expression during the cell cycle (reviewed in [1]). MuvB, consisting of the five proteins LIN9, LIN37, LIN52, LIN54 and RBBP4, associates with the p130 retinoblastoma protein paralogue and with E2F4 and DP1 to form DREAM, which represses cell cycle genes in quiescence and early G1 [2, 3]. In contrast, in S phase, the interaction of the MuvB core with p130/E2F4/DP1 is lost and MuvB now binds to the B-MYB (MYBL2) transcription factor to form the Myb-MuvB complex (also called MMB) [2, 4–6]. In late S-phase the transcription factor FOXM1 joins the complex [6, 7]. Genome wide expression studies combined with chromatin immunoprecipitation (ChIP) experiments demonstrated that MMB and MMB-FOXM1 act synergistically to activate the expression of genes whose products have important functions in mitosis and cytokinesis such as PLK1, cyclin B or NUSAP1 [6, 8]. Binding of MMB-FOXM1 to their target promoters is, at least in part, mediated by the LIN54 subunit, which contains two cysteine-rich domains that function as a DNA-binding domain to recognize conserved CHR (cell cycle homology region) DNA-motifs in the promoter region [9–12]. The functional importance of MMB is underlined by the observation that inactivation of MMB in cell lines results in mitotic defects. Furthermore mice with disrupted MuvB function die early during development due to a defect in implantation [8, 13].

FOXM1 and B-MYB are overexpressed in a wide range of different cancers [14, 15]. Furthermore, high levels of B-MYB can predict the probability of breast cancer recurrence and B-MYB is one of 21 genes that were selected for a commercial gene-expression profile test (using RT-PCR) marketed under the brand name Oncotype DX to profile early stage breast cancer [16]. In addition to B-MYB, at least three genes in the Oncotype DX test are direct target genes of MMB-FOXM1 (Aurora A, Survivin and Cyclin B1). In addition, the MuvB subunit LIN9 is part of the Mammaprint breast cancer prognostic gene signature [17]. In conclusion, high levels of B-MYB, FOXM1, LIN9 and some of their target genes are associated with a poor prognosis in different types of cancer.

Previous work in our laboratory as well as from other groups has shown that MMB function is regulated by the p53 tumor suppressor: p53 inhibits MMB and promotes the formation of DREAM, thereby resulting in reduced expression of mitotic genes [18–21]. The p53 induced formation of DREAM is, at least in part, mediated by the CDK-inhibitor p21. Conversely, in p53-mutant cells MMB-B-MYB is hyperactive resulting in increased expression of mitotic genes in these cells. Recent metaanalysis studies confirmed that hundreds of cell cycle genes are repressed by the p53-p21-DREAM pathway and that a subset of these genes are activated by MuvB, B-MYB and FOXM1 in G2/M [19, 21]. We recently demonstrated that MuvB-B-MYB is required for tumorigenesis *in vivo* in a mouse model of lung cancer and that deletion of B-MYB or of the LIN9 subunit MuvB inhibited lung tumor formation driven by oncogenic K-RAS and loss of p53 [22]. Furthermore, proliferation of lung cancer cell lines strongly depended on MuvB and B-MYB. Taken together these observations indicate that MuvB, B-MYB and FOXM1 contributes to tumor cell proliferation by activating the expression of mitotic genes.

These observations raise the question which are the critical targets downstream of MuvB, B-MYB and FOXM1 that are important for oncogenesis. Given that mitotic kinesins are frequently overexpressed in tumor cells and that certain kinesins have been identified as novel target genes of MuvB, B-MYB and FOXM1 [22–24], we sought to determine the mitotic kinesins that are directly regulated by MuvB, B-MYB and FOXM1. Kinesins are a family of ATP-dependent motor proteins that regulate the dynamic properties of microtubule [25, 26]. Of the 45 kinesins in the human genome, which are classified into 14 distinct families, at least 16 have been implicated in coordinating mitosis and cytokinesis [27]. They are responsible for the formation and function of the mitotic spindle, chromosome segregation and for cytokinesis. In tumor cells mitotic kinesins overexpression is associated with more advanced stages of the disease [26]. For example, KIFC1 expression is associated with brain metastasis of lung cancer. It is involved in centrosome clustering in cancer cells with supernumerary centrosomes [28]. Other examples are KIF4A, which is a prognostic marker for cervical cancer and non-small cell lung cancer and KIF2C, which is overexpressed in breast, gastric, colorectal and pancreatic cancer [29–31].

Little is known about the mechanisms that contribute to kinesin overexpression in cancer cells. In this study, we investigated the regulation of 15 mitotic kinesins by MuvB, B-MYB and FOXM1. We also investigated CEP55 and PRC1, two microtubule-associated non-motor proteins (MAPs) that like the mitotic kinesins also regulate the microtubule network during mitosis. We demonstrate that a) at least six mitotic kinesins as well as CEP55 and PRC1 are direct targets of MMB in MDA-MB-231 breast cancer cells, b) suppression of KIF23 and PRC1 strongly suppressed proliferation of MDA-MB-231 cells, c) gene expression levels of PRC1 and KIF23 have prognostic value in terms of survival of breast cancer patients. d) The set of MMB-FOXM1 regulated kinesins genes and 4 additional kinesins which we referred to as the mitotic kinesin signature (MKS) showed prognostic value and are linked to poor outcome in breast cancer patients. Taken together mitotic kinesins could be used as prognostic biomarker and could be potential therapeutic targets for the treatment of breast cancer.

## RESULTS

**Figure 1:**
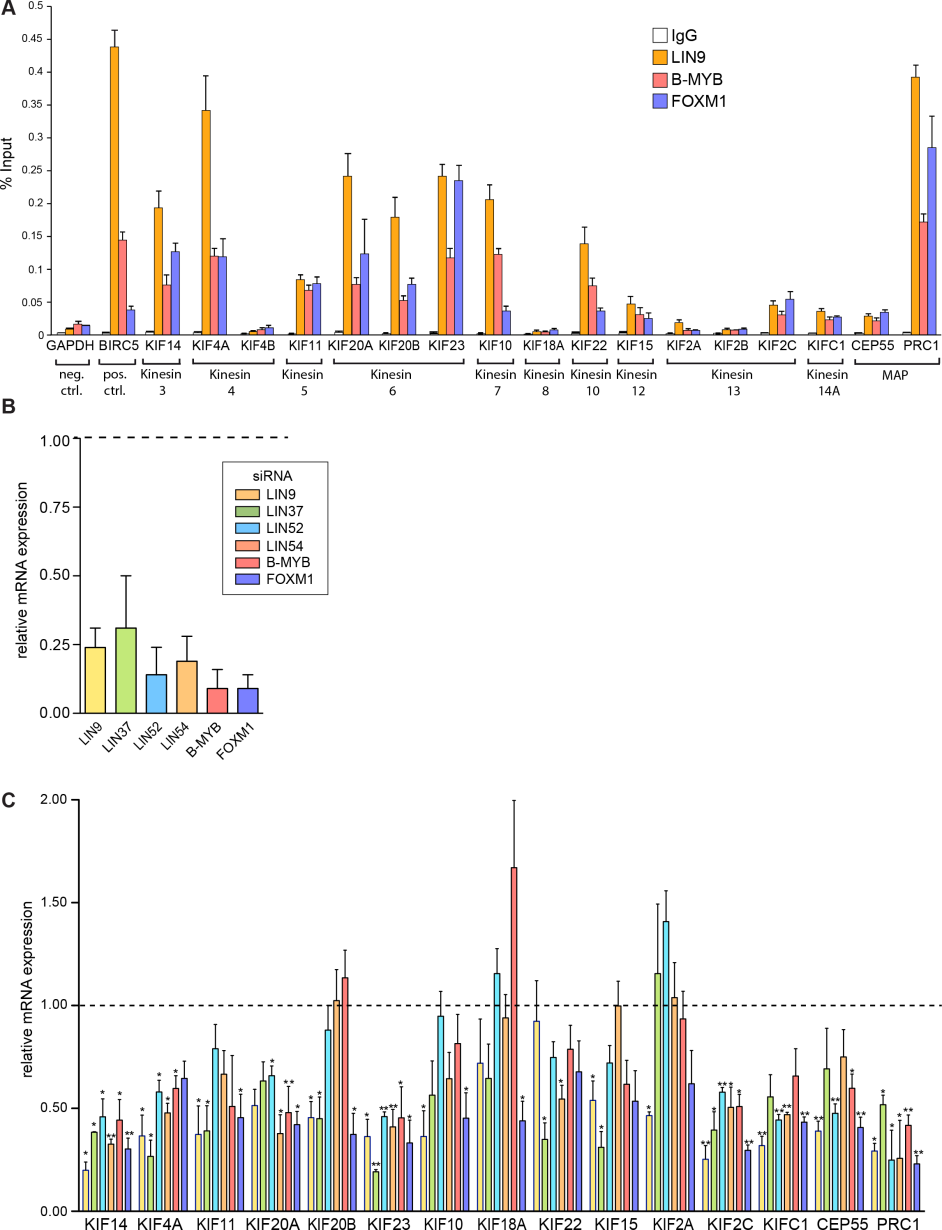
MMB and FOXM1 regulate the expression of mitotic kinesins and MAPs in breast cancer cells (**A**) ChIP assay to analyze binding of LIN9, B-MYB and FOXM1 to the promoters of 15 kinesins and to the promoters of CEP55 and PRC1 in MDA-MB-231 cells. The GAPDH2 promoter was used as a negative control and the BIRC5 promoter as positive control. Binding to the indicated promoters was analyzed by RT-qPCR. Data are mean +/– SD (*n* = 3 technical replicates). Similar results were observed in 2 independent experiments. (**B**) MMB subunits or FOXM1 were depleted by RNA interference (RNAi) in MDA-MB-231 breast cancer cells. Knockdown was validated by reverse transcriptase–quantitative PCR (RT–qPCR). Data shown are relative expression values compared with expression in cells transfected with nonspecific siRNA. (**C**) MMB subunits or FOXM1 were depleted by RNAi in MDA-MB-231 cells for 48 hours. The expression of the indicated genes was analyzed by RT-qPCR. Shown are relative expression values compared with expression in cells transfected with nonspecific siRNA and relative to the expression of the RPS14. Values are the mean +/– SD of three independent experiments. Statistical significance is indicated. *p < 0.05, **p < 0.01, Student's *t*-test.

To gain insight in regulation of mitotic kinesin expression in breast cancer cell lines by MMB and FOXM1, we performed chromatin immunoprecipitation (ChIP) assays in MDA-MB-231 breast cancer cells. We used antibodies specific for either the MuvB core subunit LIN9 or for the transcriptional activators B-MYB (MYBL2) and for FOXM1. Unspecific IgG was used as a control. qPCR analysis was used to analyze binding to the promoters of 15 kinesins with suggested functions in mitosis and cytokinesis. We also investigated binding of MMB to the promoters of CEP55 and PRC1, two microtubule-associated non-motor proteins (MAPs) that like the mitotic kinesins also regulate the microtubule network during mitosis. As expected LIN9, B-MYB and FOXM1 bound to the BIRC5 promoter, a validated MMB target gene [4], which was used as positive control (Figure 1A). The GAPDH2 promoter was used as negative control and displayed only a basal enrichment. LIN9, B-MYB and FOXM1 strongly bound to the promoters of 8 of 15 mitotic kinesins (KIF14, KIF20A, KIF11, KIF4A, KIF20B, KIF23, KIF10, KIF22) and to the PRC1 promoter. All three proteins also moderately bound to the promoters of CEP55, KIF15, KIF2C and KIFC1. Consistent with these data, CHR elements have previously been identified in the promoters of mitotic kinesins [32] and the mitotic kinesins were predicted to be targets of MMB and FOXM1 in a recent meta-analysis [19]. Although binding of MMB and FOXM1 to the KIF18 promoter has previously been observed in T98G cells, and a CHR element has been identified in its promoter, no binding was detected in MDA-MB-231 cells [6, 32]. In addition, no binding of MMB and FOXM1 to the promoters of KIF4B, KIF2A and KIF2B was detected. These kinesins have not previously been described as targets of MMB and FOXM1, which is consistent with our ChIP data.

To further test whether mitotic kinesins and MAPs are transcriptionally regulated by MMB-FOXM1 in breast cancer cells, we depleted MMB subunits in MDA-MB-231 cells by RNAi and analyzed changes in expression of mitotic kinesins by RT-qPCR. First, efficient depletion of the MuvB subunits LIN9, LIN37, LIN52, LIN54, and of B-MYB and FOXM1 by the respective siRNA was confirmed by RT-qPCR (Figure 1B). Next, expression of 13 mitotic kinesins and of CEP55 and PRC1 was analyzed. KIF4B and KIF2B were not investigated because they were not expressed at detectable levels in MDA-MB-231 cells. The expression of six mitotic kinesins (KIF14, KIF4A, KIF20A, KIF23, KIF2C and KIFC1) was significantly inhibited by depletion of at least 4 of the 6 FOXM1/MMB subunits (Figure 1C). Similarly, CEP55 and PRC1 were also regulated by MMB and FOXM1. The other seven investigated mitotic kinesins were not affected by depletion of B-MYB and were only downregulated by inhibition of one or two subunits of MuvB, indicating that they are not targets of Myb-MuvB in MDA-MB-231 cells.

The lack of regulation of KIF18A and KIF2A by MMB-FOXM1 is consistent with the results from the ChIP assays in which no binding of MMB and FOXM1 to their promoters was detected. However, the expression of the remaining five mitotic kinesins (KIF11, KIF20B, KIF10, KIF22 and KIF15) was independent of MMB-FOXM1, despite binding of LIN9, B-MYB and FOXM1 to the promoters of these genes.

Together with the ChIP data, this indicates that at least six out of 15 investigated mitotic kinesins are direct targets of MMB and of FOXM1 in breast cancer cells. In addition, the two investigated MAPs, CEP55 and PRC1, are also direct targets of MMB and FOXM1 in MDA-MB-231 cells. On the other hand, the expression of seven mitotic kinesins is independent of MMB and FOXM1 and two mitotic kinesins are not expressed in MDA-MB-231 cells.

Interestingly, PRC1, CEP55 and four out of six mitotic kinesins regulated by MMB and FOXM1 are key factors involved in assembly and organization of the central spindle and in midbody function. The central spindle is formed after anaphase onset and consists of antiparallel microtubule bundles and plays an important role determining the cleavage plane in anaphase and telophase and in formation of the actomyosin-based contractile ring that mediates cytokinesis [33, 34]. In telophase the contractile ring compresses the central spindle to form the midbody which serves as a platform for the recruitment of proteins that mediate abscission. PRC1 has been proposed to act as central spindle matrix protein and receptor for the kinesins KIF14, KIF4A, KIF20A and KIF23 that bundle spindle microtubule, control RhoA activity and regulate the activity of the mitotic kinases PLK1 and Aurora B [35]. In late telophase the coiled-coil protein CEP55 is recruited to the midbody by KIF23 to activate the abscission process [36]. The other two mitotic kinesins identified as direct targets of MMB-FOXM1 function earlier during mitosis: KIFC1, a member of the kinesin 14 family, is involved in bipolar spindle formation. In cancer cells it is required for centrosome clustering in cells with supernumerary centrosomes [28]. KIF2C plays an important role in chromosome segregation in anaphase and the correction of incorrect kinetochore-microtubule interactions.

**Figure 2:**
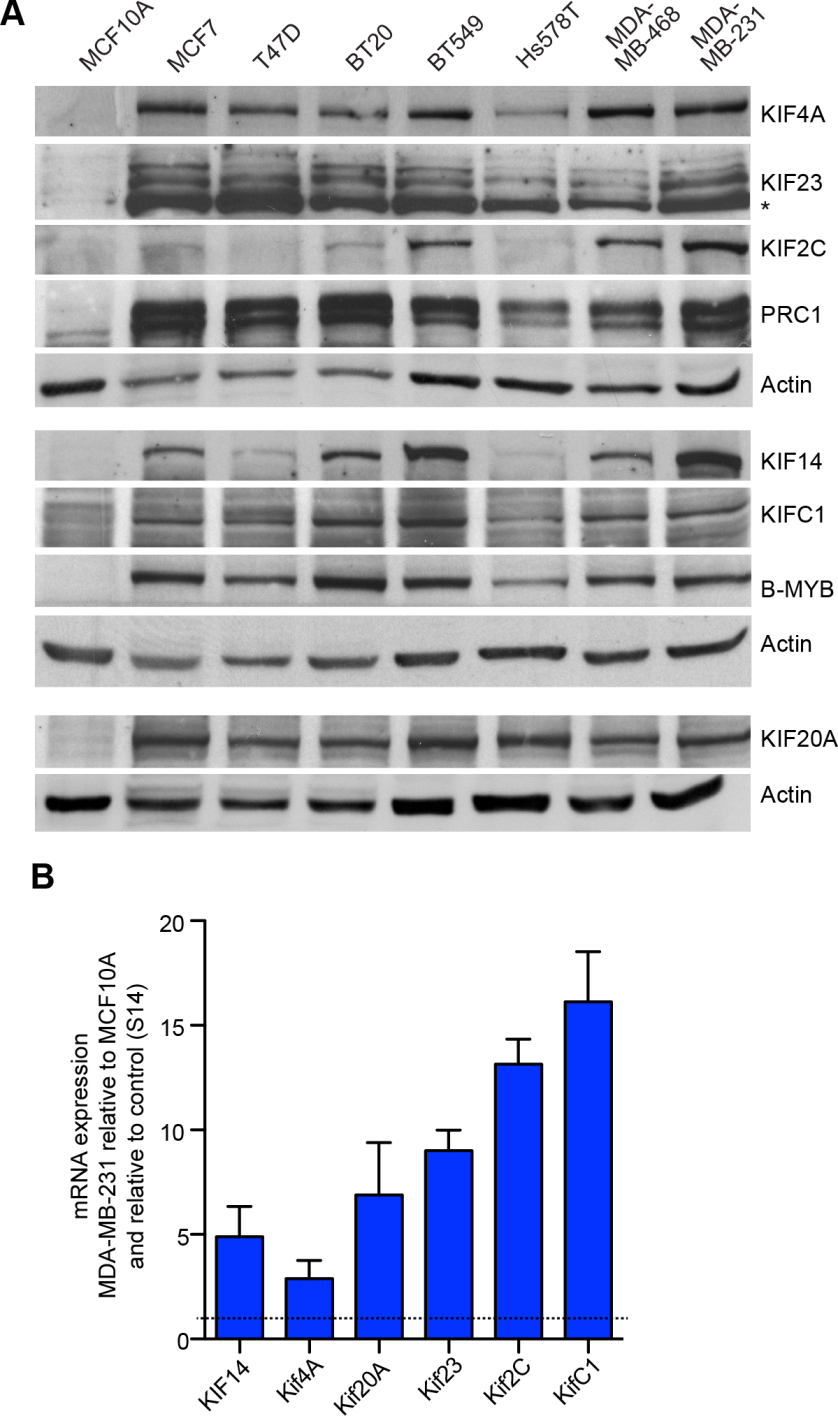
Elevated expression of mitotic kinesins in breast cancer cell lines (**A**) Expression of six selected mitotic kinesins and of PRC1 in a panel of breast cancer cell lines compared to MCF10A control cells was analyzed by immunoblotting. Actin was used as a loading control for the corresponding blots. (**B**) mRNA expression of six selected kinesins and PRC1 was analyzed in MCF10A cells and in MDA-MB-231 cells by RT-qPCR. Expression differences were calculated relative to RPS14. Values are the mean +/– SD of three different experiments.

We next focused on the six mitotic kinesins and on PRC1 that were identified as direct targets of MMB and FOXM1 in ChIP and RNAi assays. We first investigated their expression in a panel of different breast cancer cell lines (MCF7, T-47D, BT-20, BT-549, Hs 578T, MDA-MB-468, and MDA-MB-231) by immunoblotting. As a control, we used untransformed MCF10A mammary gland epithelial cells. We found that KIF23, KIF20A, KIF4A, KIFC1 and PRC1 were expressed at high levels in all investigated breast cancer cell lines. KIF14 was elevated in all cancer cell lines except for Hs578T and T47D. Finally, KIF2C was elevated in BT549, MDA-MB-468 and MDA-MB-231 cells (Figure 2A). B-MYB was also elevated in the breast cancer cell lines, consistent with its role in regulating kinesin expression.

The upregulation of mitotic kinesins in tumor cell lines could be due to protein stabilization or due to increased mRNA expression. To discriminate between these possibilities, we analyzed mRNA expression of kinesins by RT-qPCR. mRNA expression of kinesins was very high in MDA-MB-231breast cancer cells compared to normal untransformed MCF10A cells. This observation confirms that mitotic kinesins are up-regulated on the transcriptional level. Together with the data shown in Figure 1, this suggests that B-MYB and FOXM1 in conjunction with the MuvB complex contribute to the overexpression of mitotic kinesins and MAPs in breast cancer cells.

**Figure 3:**
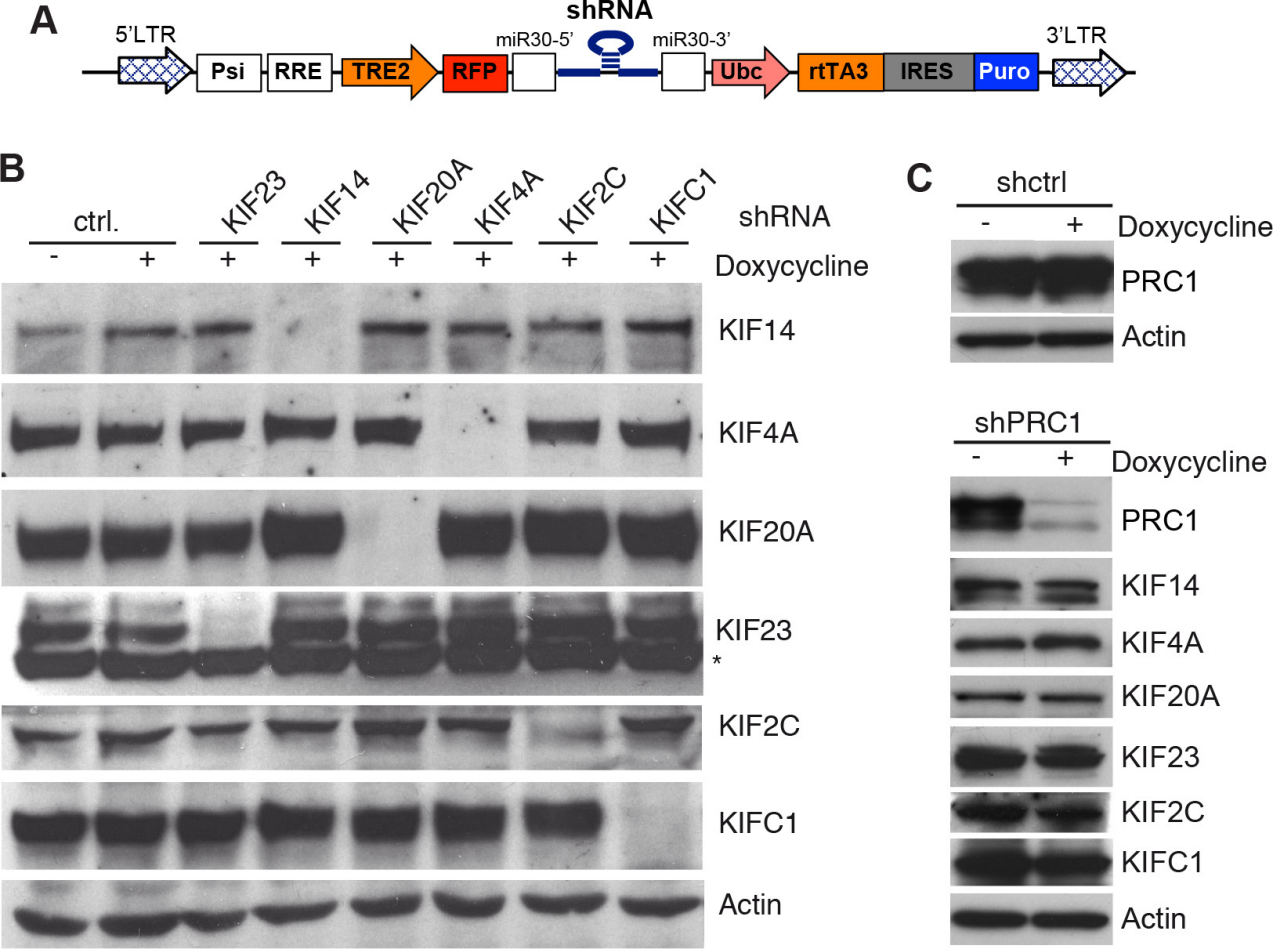
Inducible knockdown of selected mitotic kinesins and PRC1 in MDA-MB-231 cells (**A**) Scheme of the pINDUCER vector for doxycycline-inducible expression of shRNAs. (**B**) Stable MDA-MB-231 cells expressing the indicated inducible shRNAs directed at selected mitotic kinesins were generated. Knockdown of the target of the shRNA after induction of the shRNA with doxycycline for 48 hours was determined by immunoblotting. Actin was used as a loading control. (**C**) Stable MDA-MB-231 cells expressing a shRNA specific for PRC1 were generated. Expression of PRC1 and the indicated kinesins after induction of the shRNA with doxycycline for 48 hours was determined by immunoblotting. Actin was used as a loading control.

Although these observations suggest that MMB-FOXM1 might be a therapeutic target for the treatment of breast cancer, components of the MMB-FOXM1 complex are not druggable due to the lack of enzymatic activity. We therefore asked whether the six kinesins regulated by MMB-FOXM1 represent possible therapeutic targets. Because inhibition of MMB-FOXM1 inhibits proliferation and results in mitotic defects, we assessed whether the identified six mitotic kinesins partially contribute to this function in MDA-MB-231 cells. To address this question we first cloned doxycycline-inducible small hairpin RNAs (shRNAs) directed at mitotic kinesins into the lentiviral pINDUCER vector (Figure 3A) [37]. Next, lentiviral supernatants were produced in HEK293 cells and used to infect MDA-MB-231 cells to generate stable MDA-MB-231 cells expressing these shRNAs. After selection, the knockdown of mitotic kinesins was analyzed by immunoblotting (Figure 3B). Upon doxycycline-induction for 48 hours, the respective targeted protein was specifically and strongly downregulated without inhibiting the expression of other mitotic kinesins. Of note, KIF20A expression was increased after the depletion of KIF14, KIF4A, KIF2C and KIFC1 (Figure 3B). We also generated MDA-MB-231 cells stably expressing a shRNA specific for PRC1 and confirmed that depletion of PRC1 does not affect expression of kinesins (Figure 3C).

**Figure 4:**
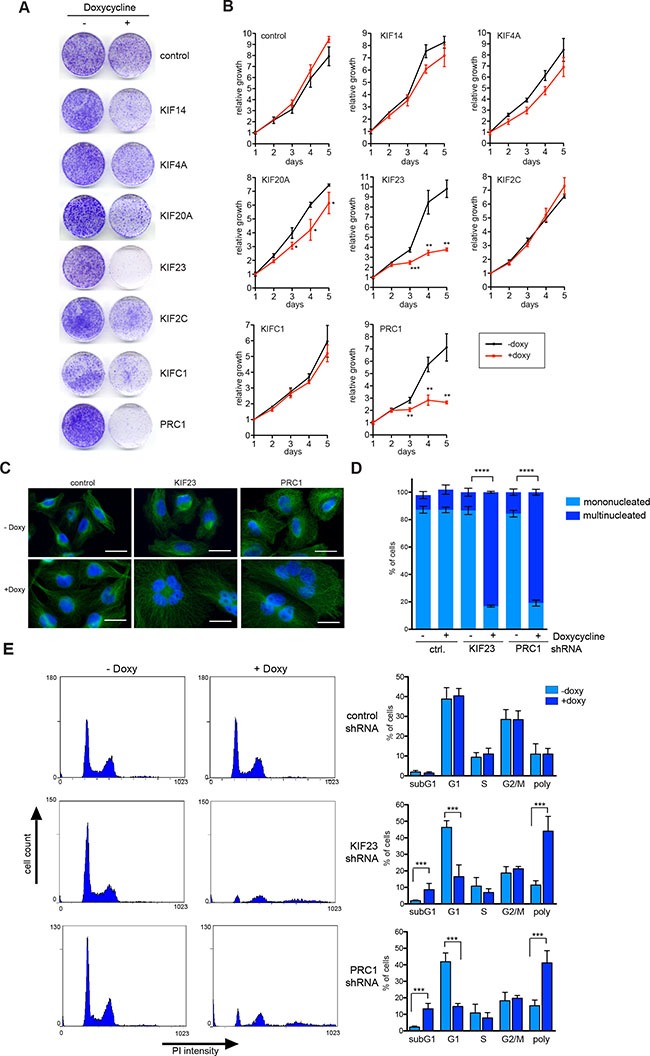
KIF23 and PRC1 are required for proliferation of MDA-MB-231 cells (**A**) Colony formation assay. MDA-MB-231 cells stably transduced with the indicated inducible shRNA constructs were plated at low density. Colony formation in absence and presence of doxycycline (to induce the shRNA) was determined by crystal violet staining after 10 days. (**B**) Growth of MDA-MB-231 cells stably expressing the indicated kinesin-specific shRNAs in presence or absence of doxycycline was analyzed over 5 days. Mean of three independent replicates. Error bars represent standard deviation. Significance levels versus control are indicated. *p < 0.05; **p < 0.001, ***p < 0.0001; Student's *t*-test. (**C**) MDA-MB-231 expressing inducible shRNAs direct at KIF23 and PRC1 were treated with or without doxycycline for 72 hours. Subsequently the cells were fixed and immunostained with tubulin (green) and Hoechst (blue) and investigated by fluorescence microscopy. Bar: 25 μm. (**D**) Quantification of mononucleated and multinucleated cells upon depletion of KIF23 or PRC1 in MDA-MB-231 cells or after induction of a control shRNA. Error bars represent standard deviation of three biological replicates. Statistical significance is indicated. **p < 0.01, Student's *t*-test. (**E**) The cell cycle profiles of KIF23 and PRC1 depleted cells by doxycycline treatment for 72 hours and of control cells was analyzed by flow cytometry. The percentage of cells in the different phases of the cell cycle was determined. Mean of four different experiments and standard deviation. Statistical significance is indicated. ***p < 0.001, Student's *t*-test.

Next, conditional MDA-MB-231 knockdown cells were plated at low density and treated without or with doxycycline to induce the expression of the shRNA. The number of colonies was determined after 10 days by crystal violet staining (Figure 4A). Importantly, when MDA-MB-231 cells stably infected with a vector with a control shRNA were treated with doxycycline, colony formation was not inhibited (Figure 4A). In contrast, depletion of the investigated mitotic regulators inhibited colony formation with different efficiency (Figure 4A). Strongest growth inhibitory effects were observed after inhibition of KIF23 and PRC1. The other shRNAs tested had moderate growth inhibitory effects in MDA-MB-231 cells. Quantitative growth assays confirmed that the depletion of KIF23 and PRC1 strongly inhibited the proliferation of MDA-MB-231 cells (Figure 4B).

Because MDA-MB-231 cells were strongly dependent on KIF23 and PRC1, a more detailed analysis of their function was performed next. Microscopic examination and quantification showed that silencing of KIF23 and PRC1 dramatically increased the number of bi – and multinucleated cells (Figure 4C, 4D), consistent with their known function in mitosis and cytokinesis. Fluorescence-activated cell sorting confirmed dramatic polyploidization following KIF23 and PRC1 depletion (Figure 4E). The sub G1 population, indicative of degraded DNA, a hallmark of apoptosis, was also increased following KIF23 or PRC1 depletion (Figure 4E). In summary, our results indicate that proliferation of MDA-MB-231 strongly depends on KIF23 and PRC1, suggesting that these two proteins could be potential targets for therapy.

**Figure 5:**
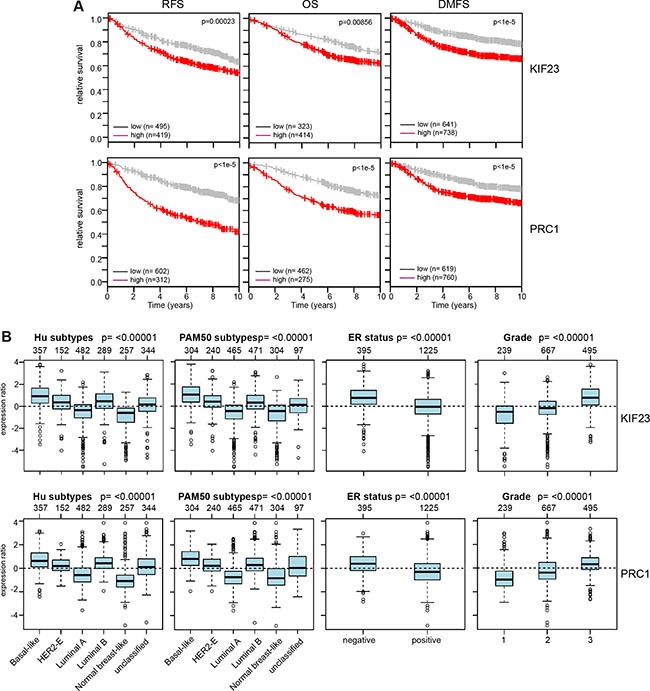
Clinical relevance of KIF23 and PRC1 expression in breast cancer (**A**) Kaplan-Meier plots showing relapse-free survival or overall survival of breast cancer patients based on expression of KIF23 or PRC1. RFS: relapse free survival; OS: overall survival; DMSF: distant metastasis free survival. (**B**) Expression of KIF23 and PRC1 across different breast cancer tumor subtypes (Hu and PAM50), according to ER status and in breast cancers of different histological grade (1, 2 and 3). Boxplots show mean centered Log_2_ expression values. The analysis was performed with the Gene Expression-Based Outcome for Breast Cancer Online web tool (GOBO).

To determine whether these findings are of clinical significance, we asked whether expression of KIF23 and PRC1 is associated with prognosis of breast cancer by examining previously published microarray data sets. Strikingly, tumors with high expression of KIF23 or PRC1 showed a significant shorter relapse-free survival (RFS), overall survival (OS) and distant metastasis free survival (DMSF) compared to tumors with low expression of these genes (Figure 5A). We next examined expression of KIF23 and PRC1 in breast cancer gene expression datasets grouped according to HU and PAM50 subtypes (Figure 5B). Both genes were expressed at higher levels the more aggressive tumor subtypes basal-like and luminal B (Figure 5B). Furthermore, a similar analysis showed that both KIF23 and PRC1 are expressed at higher levels in more aggressive grade 3 tumors compared to grade 1 and grade 2 tumors and in ER-negative versus ER-positive tumors.

**Figure 6:**
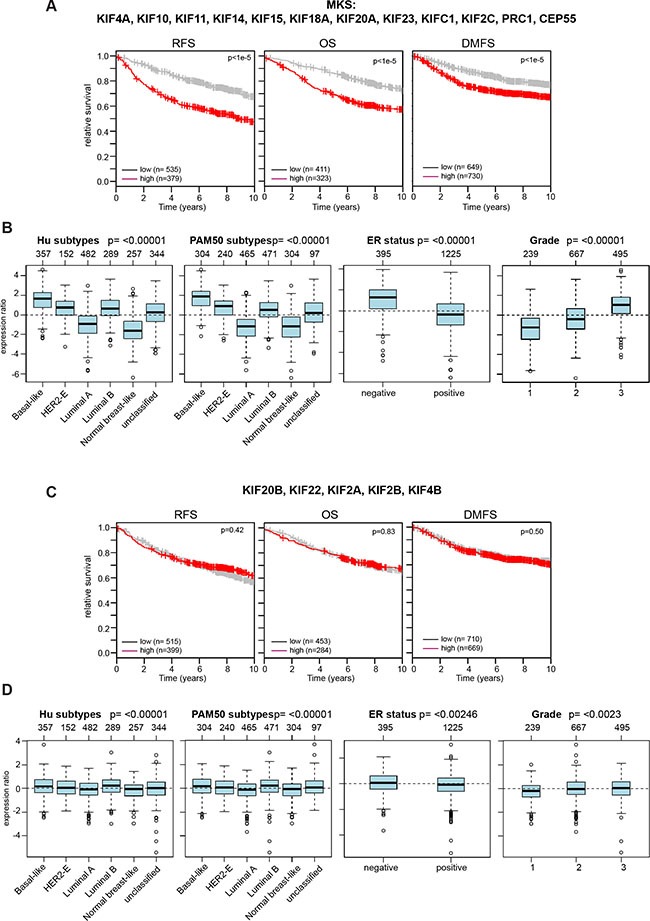
Expression of the mitotic kinesins signature (MKS) is linked to clinical outcome of breast cancer patients and is enriched in specific cancer subtypes (**A**) Kaplan-Meier plots showing relapse-free survival or overall survival of breast cancer patients based on expression of the MKS. (**B**) Expression of MKS genes in cancer subtypes (Hu and PAM50), according to ER status and in breast cancers of different histological grade (1, 2 and 3). Boxplots show mean centered Log_2_ expression values. The analysis was performed with the Gene Expression-Based Outcome for Breast Cancer Online web tool (GOBO). (**C** and **D**) Expression of the indicated mitotic kinesins is not linked to the survival of breast cancer patients and is not strongly enriched in cancer subtypes.

We next interrogated microarray data for a link between the expression of additional mitotic kinesins and the survival of breast cancer patients. This analysis resulted in a list of 12 genes linked to the prognosis of breast cancer which we referred to as the mitotic kinesin signature (MKS). MKS contains the two investigated MAPs (CEP55 and PRC1) and all six mitotic kinesins regulated by MMB and FOXM1. In addition, four additional mitotic kinesins (KIF10, KIF11, KIF18A, KIF15) that were independent of MMB and FOXM1 were also included in the signature. Significantly higher expression of MKS genes was correlated with worse RFS, OS and DMSF in breast cancer patients (Figure 6A). Furthermore, the MKS proteins were expressed at higher levels in more aggressive subtypes (basal-like, luminal B, ER-negative and grade 3 tumors) (Figure 6B). In stark contrast, expression of the remaining 5 mitotic kinesins that are not regulated by MMB and FOXM1 was not correlated with survival and their expression did not strongly differer in specific breast cancer subtypes (Figure 6C, 6D).

## DISCUSSION

Our study addressed the regulation of mitotic kinesins in breast cancer cells. Insights into the regulation of kinesin expression are important for the development of therapeutic approaches to target kinesin expression in cancer cells. By RNAi and ChIP, we demonstrate that at least six mitotic kinesins are direct targets of the MuvB core, B-MYB and FOXM1. It is interesting to note that four of the six kinesins regulated by MMB and FOXM1, namely KIF4A, KIF14, KIF20A and KIF23, are involved in formation and function of the central spindle (also called spindle midzone), the region of overlapping microtubules in the center of the spindle after the chromosomes have separated [38]. The expression of two microtubule-associated proteins, PRC1 and CEP55, which are also critical for the function of the central spindle and the midbody is also under control of MMB and FOXM1. The central spindle has essential functions in cytokinesis and abscission by mediating the activation of the small GTPase RhoA at the cell cortex and the cleavage furrow and by promoting contractile ring formation [34]. The midbody is important for the recruitment of proteins that mediate abscission, the final stage of cell division. The two other kinesins regulated by MMB and FOXM1 in MDA-MB-231 cells, KIFC1 and KIF2C, function earlier during mitosis in bipolar spindle formation and chromosome segregation.

The six kinesins and two MAPs regulated by MMB and FOXM1 are expressed at higher levels in breast cancer cell lines compared to control cell lines. Interrogation of breast cancer gene expression datasets showed that this set of genes and 4 additional kinesins which we referred to as the mitotic kinesin signature (MKS) showed prognostic value and are linked to poor outcome in breast cancer patients (Figure 6). Although higher expression levels of MKS genes could be a consequence of the high proliferation rate of cancer cells, a subset of the MKS genes could play a causal role in tumor initiation or progression. Overexpression of proteins important for central spindle formation in cancer cells likely alters spindle dynamics, chromosome segregation and cytokinesis. This could promote chromosomal instability (CIN) and aneuploidy, which is tightly associated with tumorigenesis [39, 40]. In support of this notion, the mitotic kinesins KIF20A, KIF4A, and PRC1 and CEP55 are part of chromosomal instability signature that is correlated with poor clinical outcome [41]. In addition, mitosis independent functions for kinesins and MAPs that contribute to tumorigenesis have also been observed. For example, activation of the ß-catenin dependent transcription by PRC1 promoting proliferation, stemness and metastasis of hepatocellular carcinoma has recently been reported [42]. KIF14 activates AKT signaling, which contributes to chemoresistance of triple negative breast cancer [43].

The finding that the MMB-FOXM1 complex regulates the expression of a set of mitotic kinesins relevant for breast cancer prognosis suggest that it might be a possible therapeutic target. However, it will be difficult to inhibit components of the MMB-FOXM1 because no enzymes have so far been identified in the complex. Mitotic kinesins on the other hand are considered druggable because of their enzymatic activity [44]. Due to their specialized functions in mitosis, kinesin inhibition might produce fewer side effects than other anti-mitotic drugs such as drugs that target tubulin. An example for a mitotic kinesin that is already investigated in phase I and II clinical trials as a cancer target is the kinesin Eg5 (also called KIF11 or KSP) [45]. Eg5 functions in early stages of mitosis and is involved in bipolar spindle formation during prometaphase. Its inhibition can induce mitotic catastrophe and apoptotic cell death in cancer cells.

Silencing of KIF23 expression strongly suppressed proliferation of MDA-MB-231 cells due to multinucleation and polyploidization, suggesting that KIF23 is a potential target for therapy of breast cancer. Depletion of the other investigated kinesins moderately suppressed proliferation of MDA-MB-231 cells. It is possible that simultaneously blocking multiple kinesins will have stronger effects on proliferation. Although in this study we focused on the regulation of kinesins in breast cancer, the results are likely also relevant for additional tumor types. For example, the depletion of KIF23 has been shown to inhibit glioma cell proliferation [46]. In addition, we have recently found that KIF23 is regulated by p53-MuvB-B-MYB in lung cancer cell lines *in vitro* and that KIF23 is required for lung tumorigenesis in a mouse model of non-small cell lung cancer *in vivo* [22].

In summary, we identified six mitotic kinesins and two MAPs as direct transcriptional targets of MMB-FOXM1. These genes and four additional kinesins are linked to the prognosis of breast cancer patients. Inhibition of KIF23 and PRC1 had strong antiproliferative effects in breast cancer cells. KIF23 and PRC1 could be used as prognostic biomarker and could be potential therapeutic targets for the treatment of breast cancer.

## MATERIALS AND METHODS

### Cell culture

T47D, Hs578T and BT549 cells were cultured in RPMI containing 10% FCS (Life Technology) and 1% penicillin/streptomycin. MCF7, BT20, MDA-MD-468 and MDA-MD-231 cells were cultured in DMEM supplemented with 10% FCS (Life Technology) and 1% penicillin/streptomycin. MCF10A cells were cultured in DMEM/F12 with 1% penicillin/streptomycin, 5% horse serum (Sigma), 20 ng/ml EGF, 100 ng/ml cholera toxin, 0.01 mg/ml insulin and 500 ng/ml hydrocortisone.

### RNAi

The siRNAs targeting LIN9, LIN37, LIN52, LIN54, B-MYB and FOXM1 have been described before [2]. Double stranded RNA was purchased from MWG. siRNAs were transfected in a final concentration of 50 nM using RNAiMAX (Invitrogen) according to the manufacturer's protocol. For inducible expression of shRNAs in MDA-MB-231 cells, the lentiviral pINDUCER10 vector was first modified by replacing the puromycin resistance gene for a blasticidin resistance gene. Next, mir30 based shRNAs were cloned into the vector. The targeting sequences were:

KIF14: 5′ CCAGAGCAAGTTGGATATCAAA 3′

KIF4A: 5′ CGCCGAGATAGAGACAGAGTTA 3′

KIF20A: 5′ CACGCAAGAACCTGCTATCAGA 3′

KIF23: 5′ CCCAGAGTTTGCAGATATGATA 3′

KIF2C: 5′ CCAGGAAGCAGACTCAAGAGGA 3′

KIFC1: 5′ ATAGTGCTAAGATGCTCATGTT 3′

PRC1: 5′ CGACGACCATCTTGCAACTAGA 3′

MDA-MB-231 cells lines stably expressing the shRNA were generated by lentiviral infection and selection with 10 μg blasticidin for 7 days. To induce the shRNA, cells were treated with 1 μg/ml doxycycline for the indicated time points.

### Antibodies

The following primary antibodies were used: Actin: sc-47778 (Santa Cruz Biotechnology). LIN9: ab62329 (Abcam). B-MYB: sc-724 (Santa Cruz Biotechnology) and LX015.1 (kind gift from Roger Watson (Tavner et al., 2007). FOXM1: sc-502 (Santa Cruz Biotechnology), KIF14: A300-912A (Bethyl Laboratories). KIF4A: A301-074A (Bethyl Laboratories). KIF20A: A300-879A/Bethyl Laboratories). KIF23: sc-867 (Santa Cruz Biotechnology). KIF2C: kind gift from Linda Wordeman. KIFC1: 12313 (Cell Signalling). PRC1: sc-8356 (Santa Cruz Biotechnology). α-tubulin (Sigma T6074).

### Immunoblotting

Cells were lysed in TNN [50 mM Tris (pH 7.5), 120 mM NaCl, 5 mM EDTA, 0.5% NP40, 10 mM Na_4_P_2_O_7_, 2 mM Na_3_VO_4_, 100 mM NaF, 10 mg/mL phenylmethylsulfonyl fluoride, protease inhibitors (Sigma)]. Proteins were separated by SDS-PAGE, transferred to PVDF membrane and detected by immunoblotting.

### Chromatin immunoprecipitation (ChIP)

Cells were cross-linked with 1% formaldehyde for 10 min at room temperature. The reaction was stopped by adding 125 mM glycine. Cells were lysed for 10 min in lysis buffer [5 mM PIPES (pH 8.0), 85 mM KCl, 0.5% NP40, protease inhibitors (Sigma)]. Nuclei were lysed in nuclei lysis buffer [50 mM Tris (pH 8.1), 10 mM EDTA, 1% SDS, protease inhibitors (Sigma)]. Chromatin was sonicated to an approximate length of 250 to 500 bp, diluted 1:10 with dilution buffer [0.01% SDS, 1.1% Triton, 1.2 mM EDTA, 16.7 mM Tris (pH 8.2), 167 mM NaCl, protease inhibitors (Sigma)] and used for immunoprecipitation overnight. Immmunoprecipitates were collected with protein G–dynabeads (Life Technologies) for 1 h (blocked with 1 mg/mL BSA and 0.3 mg/ml ssDNA). Beads were washed seven times with LiCl washing buffer [0.25 mM LiCl, 0.5% NP40, 0.5% sodium deoxycholate, 1 mM EDTA, 10 mM Tris (pH 8.0), protease inhibitors (Sigma)] and eluted with elution buffer [50 mM Tris (pH 8.0), 1% SDS, 10 mM EDTA]. The cross-link was reversed overnight with 0.2 mM NaCl at 65°C. After proteinase K incubation for 2 h at 55°C, chromatin was purified using Qiagen DNA purification spin columns and eluted in 50 μl. Chromatin (1 μl) was used as template for quantitative real-time PCR. Primer sequences are listed in Supplementary Table 1.

### Flow cytometry

For flow cytometry, cells were fixed in 80% ethanol, DNA was stained with 69 mM propidium iodide in 38 mM sodium citrate and 100 mg/ml RNase A for 30 min at 37°C. Samples were analyzed on a Beckman Coulter Fc500.

### Proliferation assays and immunostaining

For growth curves, cells were plated in 24-well plates and expression of the shRNA was induced with 1 μg/ml doxycyline. At the indicated time points, cells were fixed in 10% formaline and stained with crystal violet. The dye was extracted with 10% acetic acid and the optical density was determined.

### Immunostaining

Cells were fixed in 3% paraformaldehyde, 2% sucrose in PBS, permeabilized with 0.2% Triton X-100 and blocked with 5% goat serum for 30 minutes. Coverslips were incubated for 1 hour at room temperature with anti-tublin antibody. Secondary antibody (α-mouse Alexa Fluor 488, Thermo Fischer) was diluted 1:500 in PBS and incubated 2 h at room temperature. Coverslips were stained with 1 μg/ml 4′,6-diamidino-2-phenylindole (Hoechst; Sigma) and mounted in IMMU-MOUNT. Cells were investigated by fluorescence microscopy and the fraction of mono- and multinucleated cells was determined.

### RNA isolation, reverse transcription and quantitative real-time PCR

Total RNA was isolated with Total RNA Isolation Reagent (Thermo Scientific). 1 μg RNA was transcribed using 125 units MMuLv (Thermo Scientific). Quantitative real–time PCR reagents were from Thermo Scientific and real-time PCR was performed using the Mx3000 (Stratagene) detection system. Expression differences were calculated as described before. Primer sequences are listed in Supplementary Table 1.

### Statistical analysis

Statistical analyses were performed using Prism 5 (GraphPad Software). Statistical significance was determined using Student's *t*-test. *P* values < 0.05 were considered statistically significant.

### Survival analysis

Survival analyses and gene expression correlation analysis for human breast cancer patients were obtained using the Gene Expression-Based Outcome for Breast Cancer Online web tool (GOBO) [47] (http://co.bmc.lu.se/gobo).

## SUPPLEMENTARY MATERIALS FIGURES AND TABLES




