# Supplementary material for: Central spindle proteins and mitotic kinesins are direct transcriptional targets of MuvB, B-MYB and FOXM1 in breast cancer cell lines and are potential targets for therapy
Source: Oncotarget. 2017 Jan 3;8(7):11160–72. doi: 10.18632/oncotarget.14466 (PMC5355254; doi:10.18632/oncotarget.14466)
Supplement: Supplementary file 1 [file oncotarget-08-11160-s001.pdf]

Central spindle proteins and mitotic kinesins are direct transcriptional targets of MuvB, B-MYB and FOXM1 in breast cancer cell lines and are potential targets for therapy

Supplementary Materials

Supplementary Table 1: Sequences of primers for ChIP and for RT-qPCR.

See Supplementary_Table_1
