# Supplementary material for: Central spindle proteins and mitotic kinesins are direct transcriptional targets of MuvB, B-MYB and FOXM1 in breast cancer cell lines and are potential targets for therapy
Source: Oncotarget. 2017 Jan 3;8(7):11160–72. doi: 10.18632/oncotarget.14466 (PMC5355254; doi:10.18632/oncotarget.14466)
Supplement: Supplementary file 2 [file oncotarget-08-11160-s002.docx]

**Supplementary Table S1:** Sequences of primers for ChIP and for RT-qPCR

Primers for ChIP:

| **Gene** | **fw primer (5' > 3')** | **bw primer (5' > 3')** |
| --- | --- | --- |
| BIRC5 | CCATTAACCGCCAGATTTGA | GCGGTGGTCCTTGAGAAAG |
| CEP55 | TCCTTTGTGAAATCCCGTTG | GATTCTCCCTGTGTGAAGTGC |
| GAPDH2 | GGCAGCAAGAGTCACTCCA | TGTCTCTTGAAGCACACAGGTT |
| KIF10 | GAGGGTCCTGGCCATTTT | CCCCCTACCTGCTGTTCA |
| KIF11 | GCTCCAGTGAGGATACTGCAT | CCCAGAAACTAAGCAACGACTC |
| KIF14 | TTCAAATTGCGGCCTTCT | ACCCCCTCGACACAGTCC |
| KIF15 | CCCTCCAAGTAGAGGCAAAAG | CTTCGCCAGTCGTGTTTCTT |
| KIF18A | ACCCAGAACCCATTAATCCA | GCCTATGCCCTCTGCTAGG |
| KIF20A | AAATAGTGTACCGGCGTTGG | TTTGTCTTAAGGGGAAAAACGA |
| KIF20B | TACTCCCAGCGTTCAGTGC | TGCGATGTTACCGTTTTCAA |
| KIF22 | CGGCGCGTAGTCTTGATT | CCATTCCACTCCCTCCTTG |
| KIF23 | CCTAACGTCCCGCAGTCTT | GCCTCGTACTCACGCTGAC |
| KIF2A | CCGGATCCCGTACTGTCA | GTGGTTTCAGGGGCTTTCTA |
| KIF2B | GTTCTTTCTCGCCATGATCC | AGTGAGGGTGAAGGGGTTG |
| KIF2C | ATCCCAGAGCCCTCATTGT | ACCTTGTACTGCGGGGTTC |
| KIF4A | TGAAACTTGGCGGTTAAAGC | CCCCGTAACTCACCAAACTAA |
| KIF4B | GGTGTCTCTGGGAGGGATTT | AATGTCCCTTCCCCGTTC |
| KIFC1 | ACCAGGAGGCGCTAGTCC | ACTGGCCAAGCGAAACTG |

Primers for RT-qPCR:

| **Gene** | **fw primer (5' > 3')** | **bw primer (5' > 3')** |
| --- | --- | --- |
| FOXM1 | ACTTTAAGCACATTGCCAAGC | CGTGCAGGGAAAGGTTGT |
| GAPDH | GCCCAATACGACCAAATCC | AGCCACATCGCTCAGACAC |
| KIF10 | AACAGGCCCAAGATACCTCA | TCCACAAGTTAAGGGTTTATTTGA |
| KIF11 | CATCCAGGTGGTGGTGAGAT | TATTGAATGGGCGCTAGCTT |
| KIF14 | CCTGTCTTTTTGCTTATGGTCAG | TCTTCACTAAATCCCATCATCG |
| KIF15 | GCTGCTGAAGCCTATCAGGT | GATGTTGATGCCACACGTCTA |
| KIF18A | AGCATGGTGCCATCCTACA | TCTGCAGTTAACGAACTGTTTGA |
| KIF20A | CGGCGACTAGGTGTGAGTAAG | GGATCCCTTGCGACATGA |
| KIF20B | AGGCTCGGAAGAGGAAGAGT | TTCAAATTAGTTCTGGGTGTAGCA |
| KIF22 | GCCCTGAGGAAGAGGAGATT | GGTCCATGCTGCTTAGCTTC |
| KIF23 | CCTAACGTCCCGCAGTCTT | AGGTTTCCGGGGTGTCTTAG |
| KIF2A | GAAAACTCTCGTACCTGCATGAT | TGTATTAAGAGTATTTTCACAGGATGC |
| KIF2C | AGGAGCATCTGGTTAACTCTGC | TCTGCCCAGAGGTTCTGC |
| KIF4A | TGGTCAGACAGCCCAGATG | TCTTCTAGCTTGGCGTTCATT |
| KIFC1 | GAAGAACGGAGGGGACTGAT | ACAGGGCTGCTTCTGATGTC |
| LIN37 | CACTGGCAAAAGGCCATC | GGTCGAACAGCTTGATCACAT |
| LIN52 | GATGACATCGACATGTTGAAAGA | CCGTGTCATCTCTCTGGACTC |
| LIN54 | GCCACATCAGCCAGTAGCTC | TAACAACCACTGGCTTTGCTT |
| LIN9 | CCCCACCACGGTTACATTAT | CGGCGACTGTCCTAATAAAGG |
| MYBL2 | TCCACACTGCCCAAGTCTCT | AGCAAGCTGTTGGTCTTCTTTGA |
